# Cash-for-care and its impact on older adults’ informal and formal long-term care use: evidence from continental European countries

**DOI:** 10.1007/s10198-026-01901-y

**Published:** 2026-03-04

**Authors:** Viktoria Szenkurök

**Affiliations:** Vienna University of Economics and Business, Vienna, Austria

**Keywords:** SHARE, Public benefits, Care allowance, Family role, Mixed responsibility, granting generosity, I11, I38, J14

## Abstract

This study examines whether receipt of cash-for-care (CFC) benefits promotes or displaces family-based informal or, conversely, formal long-term care use. Utilizing cross-national data from four waves (2015–2021) of the Survey of Health, Ageing, and Retirement in Europe (SHARE) covering Austria, Belgium, France, and Germany, generalized linear models — accounting for variations in granting of these benefits — are applied. With CFC having become increasingly widespread in those traditionally conservative-corporatist welfare states in Continental Europe, this study provides novel insights into the role of CFC and its impact on care use, while also revealing persistent country-specific heterogeneity. The results provide robust evidence that CFC facilitates informal caregiving, especially in Austria and Germany. However, identification using plausible exogenous variation in the generosity in granting care allowances across waves, countries, and regions reveals that the same effect does not apply to formal care. In contrast, the findings suggest that CFC may discourage formal care use. Yet, significant cross-country differences emerge, with Germany standing out as the only country where CFC incentivizes both informal and formal care use – including their combination – while also mitigating potential unmet needs. Offering policy-relevant insights on how to address emerging challenges resulting of demographic aging, evolving care needs, and cost control, this study highlights the crucial role of the specific design of the CFC scheme, particularly with respect to access and benefit allocation, in shaping the care mix.

**Supplementary Information:**

The online version contains supplementary material available at https://doi.org/10.1007/s10198-026-01901-y.

## Introduction

Cash-for-care (CFC) schemes are defined as publicly-funded monetary transfers (allowances) typically directly provided to older adults assessed to be in need of personal care [1]. With CFC often presenting a less costly and widely preferred alternative to the expansion of home and community-based care (in-kind benefits) [2–4], these schemes have been introduced in diverse care and welfare regimes across Europe during the 1990s and early 2000s as part of wider efforts to meet the growing demand for long-term care (LTC) services due to aging populations [5]. Despite existing similarities, CFC schemes vary in policy context, timing, and specific regulation (e.g., the extent to which individuals can freely choose between family-based informal and formal care) [6]. Particularly in countries with ‘weaker’ care policies and historically family-oriented systems, CFC schemes are considered the backbone of newly established LTC policies, supporting access to family-based care while flexibilising the organization of one’s care mix based on individual needs, preferences, and circumstances (‘free choice rationale’) [1, 6, 7]. In contrast, in countries with a longer tradition of social care, cash benefits play a comparatively minor role, as they emerged alongside other LTC policies to complement existing in-kind benefits, promote users’ autonomy and empowerment, while also controlling costs [1, 6].

At present, there is a lack of conclusive evidence about what kind of care arrangements are encouraged by CFC schemes [7]. What we do know, however, is that, on balance, CFC is comparatively more prevalent in countries representative of the conservative-corporatist welfare state model – with 2 to 7% of the older adults aged 65 and above having received CFC in 2021 [6, 8, 9]. Traditionally taking a family-based approach towards health and social policy, the present study thus aims to empirically investigate the overall impact of CFC on informal and formal care use in four of those countries — Austria, Belgium, France, and Germany — while exploring persisting cross-country differences.

Although there is emerging evidence suggesting that CFC facilitates a shift towards deinstitutionalization, leading to a reduction in residential care and increasing uptake of home care services [10], little is known about whether informal or formal home care arrangements are encouraged. According to theoretical considerations, the effect seems to work in both directions. Although CFC may allow families to either purchase care in the market or compensate informal caregivers for their opportunity cost [7, 10, 11], the ‘encouragement’ theory predicts the ‘crowding-in’ of family help, with CFC encouraging family-based care arrangements as opportunity costs arising from employment in the labour market become lower [12]. Heterogeneities across countries may persist though, not only due to differences in income levels and opportunity costs but also due to variations in the design and coverage of applied CFC schemes. In contrast to ‘encouragement’ theory, the competing ‘substitution’ theory suggests that CFC leads to a ‘crowding-out’ of the family, with older people more likely to resort to formal care when having needs. CFC protects the dependent’s wealth, which means that family members (particularly children or spouses) or the larger social network (e.g., friends) experience disincentives to provide care themselves (‘intra-family moral hazard’) [13–15]. Finally, theories around ‘mixed responsibility’ predict a rise in both informal and formal care use as a result of CFC receipt [12]. As benefit levels often remain below the actual costs of care, informal care may supplement care provided by professional caregivers rather than replacing it [6, 16].

First empirical research largely focusing on informal care, indicate mixed and, for some countries even inconclusive, findings. While Courbage et al. [17] found a negative association between CFC and informal care use in Spain, the authors highlight a positive association for Italy, suggesting that the exact configuration of CFC scheme might be important in explaining cross-country differences. Costa-Font et al. [7], however, contradicts earlier findings for Spain. Utilizing an instrumental variable approach, the authors demonstrate a positive effect of CFC on informal care use in Spain. Yet, overall, the role of CFC in both Spain and Italy, remains comparatively low: only 0.1% of the older adults in Italy and 0.6% of those in Spain received cash benefits in 2021 [9].

In the broader literature on welfare states, comparative studies focusing on different welfare regimes in Europe suggest that in societies with more generous welfare state systems (e.g., Norway), family assistance and welfare state services are complementary, reinforcing the concept of mixed responsibility [12]. However, previous studies primarily compared outcomes rather than delving into specific welfare state benefits, such as pensions or, more specifically, LTC-related benefits. Further evidence on the Netherlands and Germany, focusing on both informal and formal assistance, including tasks beyond personal care (e.g., paperwork, household chores, home repairs, gardening, transportation, shopping), indicate that formal care is more prevalent in the Netherlands, while informal help is more commonly provided in Germany [18]. Using waves 1 and 2 of the Survey of Health, Ageing, and Retirement in Europe (SHARE) — which do not include direct information on CFC receipt— Bakx et al. [18] conducted a decomposition analysis highlighting the importance of system-level factors, such as eligibility criteria and coverage generosity, in determining choices between formal and informal care.

Overall, thorough empirical investigations into whether LTC-related benefits influence the care mix remain scarce, largely due to data limitations. First existing studies – constrained either by geographical scope or their methodological approach – reveal inconsistent results. This study thus seeks to bridge gaps in two important ways: First, by utilizing 2015–2021 individual-level data from SHARE, which directly captures information on CFC benefit receipt across European countries, this study illuminates whether receipt of CFC benefits is associated with an overall increase in the use of informal and/or formal personal care. Second, it extends previous research by limiting the geographical scope to four Continental European countries rarely explored in previous literature — Austria, Belgium, France, and Germany — that share relatively similar approaches to LTC within the historical framework of conservative-corporatist welfare states. While each country has established its own distinct system, they share key similarities and exhibit comparable trends in health and social policies more generally, as highlighted in recent literature [8, 19–21]. Insights from this analysis are particularly relevant as LTC systems face common challenges, such as demographic shifts, increased female labour force participation, and evolving family and household structures (e.g., smaller households and fewer children, increased spatial mobility among younger generations). These strains likely pose a particular threat to the sustainability of family-centred LTC models, making it crucial to study the impact of existing policy measures, including CFC benefits.

This article thus contributes to the general lack of knowledge regarding the effectiveness of these benefits, providing insights into how such policies shape the care mix in conservative-corporatist welfare contexts. Following a contextual section providing information on the specifics of each CFC scheme, the Data and Methods used are described. The next section presents the Results, while the final section discusses them in the context of existing literature and concludes.

## Cash-for-care schemes in continental European countries

Despite existing similarities, a closer inspection of each CFC scheme reveals crucial differences, which ought to be outlined in this section. For details (e.g., regarding benefit levels), a descriptive overview is provided in the Appendix (see Table A.6).

### The Austrian Pflegegeld

**Fig. 1:**
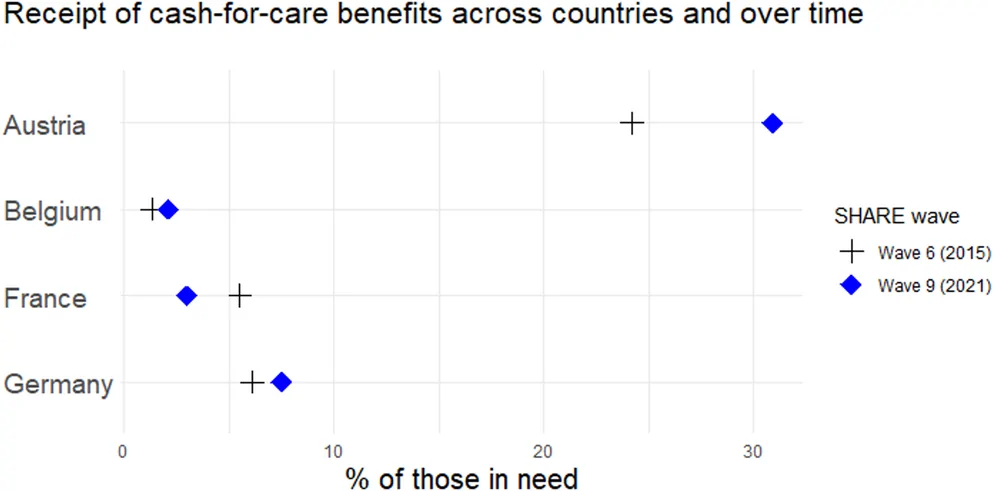
Share of older adults in need receiving cash-for-care benefits across countries in 2015 versus 2021. Note: Need is defined as having at least 1 limitation with IADL or ADL

Based on legal entitlement, Austrians were among the first in Europe to benefit from federal care allowance (*Bundespflegegeld*). Introduced in 1993, the use of CFC has become increasingly widespread since then and remains a key pillar of the present LTC system [22, 23]. To illustrate this, over the period studied, the use of CFC among older individuals in need of care increased from 24.2% in 2015 to 30.9% in 2021 (Fig. 1). Designed as a lump-sum contribution to partly cover the costs of care, care allowances are granted as a universal benefit financed through taxes (i.e., without income- or wealth-related adjustments and according to seven different care levels reflecting differing degrees of care need) [22, 24]. The specific benefit levels (outlined in Table A.6) are determined through an assessment of the time required to provide assistance with (instrumental) activities of daily living (IADL/ADL), irrespective of age. The minimum care need required for access to *Bundespflegegeld* at level 1 is 65 h per month, with the amount adjusted annually for inflation [24]. The initial assessment is usually conducted by a physician and supported by a registered nurse for reassessment [6, 22]. The care allowance is paid directly to the person in need of care, provided they are still living at home, and can be used by the recipients at their own discretion without any proof on what these benefits are spent on [24, 25].

### Care allowance(s) in Belgium

Following two major state reforms in 2001 and 2014, the competencies between the Belgian federal state, its regions, and its communities were significantly revised and redistributed. LTC in Belgium is now characterized by shared responsibilities across these levels, with a clear institutional division between the federal government and the federated entities (which include both communities and regions).

In particular, the communities (Flemish, French, and German-speaking) are primarily responsible for non-medical LTC, including the organization, regulation, and financing of most cash (and in-kind) benefits intended to support dependent persons with everyday tasks and personal care (e.g., eating, mobility, hygiene, but also instrumental activities such as meal preparation) [26–30].

Although coverage and eligibility criteria remained broadly comparable across Belgium, the former cash benefit providing assistance to older people (APA: *Allocation pour l’aide aux personnes âgées*) was regionalized in 2014 when responsibilities for non-medical LTC were devolved to the linguistic communities, resulting in distinct benefit schemes across the country. In the Brussels-Capital Region, *Iriscare* is responsible for the administration of cash benefits. In Wallonia, they are managed by the *Agence pour une Vie de Qualité* (AVIQ), while in the German-speaking community, the *Dienststelle für ein Selbstbestimmtes Leben* (DSL) is responsible [24, 25]. In Flanders, APA has been integrated into the Vlaamse Sociale Bescherming (VSB), formally established in 2016 as the umbrella framework for care allowances. Under the VSB, several benefit pillars exist, notably the *zorgbudget voor zwaar zorgbehoevenden* (for people with severe care needs, formerly known as the Vlaamse Zorgverzekering), the *zorgbudget voor mensen met een handicap*, and the *zorgbudget voor ouderen met een zorgnood*. These benefits are administered collectively by regional care funds (zorgkassen). The *zorgbudget voor zwaar zorgbehoevenden* is a monthly, non-means-tested payment for highly care-dependent individuals, both at home and in residential settings. The amount may be used at the beneficiary’s discretion [29, 30]. Other pillars of the VSB, like the budgets for persons with disabilities and older adults with care needs, have separate eligibility criteria and payment levels that may vary by income and care needs [30]. Notably, in Flanders, individuals with disabilities may receive personal budgets, which take the form of a Personal Assistance Budget (*Persoonlijke-assistentiebudget*) for minors (before the age of 21), and a Personal Budget (*Persoonsvolgend Budget*) for adults (aged 21–64 at the time of application) [31]. This budget enables individuals to employ personal assistants for daily living activities; the amount depends on the level of disability and support needed. Additionally, an informal carers’ allowance (*mantelzorgpremie*) is available in some Flemish local authorities (provinces and municipalities). Where the APA remains applicable, it is a monthly allowance that is both means-tested and needs-tested, offered to individuals aged 65 or older who meet a minimum score on a regional care-needs scale. Benefit amounts are adjusted based on income, and usage is discretionary [28].

Despite the complex nature and existing regional differences, Belgium has established a well-developed social protection system for older individuals in need of LTC, including extensive use of in-kind benefits and a comparatively stronger reliance on institutional support [24]. Correspondingly, take-up of care allowance remained at a lower level since its introduction (rising from 1.3% in 2015 to 2.1% in 2021; see Fig. 1 and Table A.4).

### The German LTC insurance (LTCI)

Since 1995, Germany’s statutory LTC insurance (LTCI) has covered the risks of LTC, providing care allowance (*Pflegegeld*) to individuals deemed in need of care, regardless of their income, to ensure adequate personal care and support measures [25]. Until the early 1990s, LTC was based on a means-tested system administered by the federal states and cities in Germany. However, increasing demand and growing financial pressure on local authorities led to a reform, introducing LTCI and recognizing the need for LTC as a universal risk [6].

Germany’s LTCI was introduced as a separate branch of the social insurance system in 1995, alongside health, accident, unemployment, and pension insurance [32, 33]. It aims at providing partial coverage of care-related costs, with individuals or families typically bearing additional expenses. The assessment of needs is based on a point-grade scheme that evaluates impairments in six key areas of life, such as mobility, self-reliance, and cognitive abilities. The total points determine one of five care levels, with higher scores indicating more severe impairments (and higher benefit levels). The care dependency of each individual is assessed independently by staff from the Medical Review Board of the Statutory Health Insurance [25]. Under the German LTCI system, individuals have the option to choose between benefits in kind or cash benefits. If chosen, a monthly amount is paid directly by the LTCI. Similar to Austria, there are no restrictions on how CFC must be spent [25, 32, 33].

Although not as significant as in Austria, care allowances have become increasingly popular in Germany in recent years, rising from 6.1% to 7.5% of community-dwelling adults aged 50 and above in need of personal care who received CFC between 2015 and 2021 (Fig. 1).

### Care allowance in France

Introduced in 2002, in France, the primary source of cash benefits for older adults arises from the universal tax-based *allocation personnalisée d’autonomie* (APA), a successor to the pre-existing *prestation specifique dependance* (PSD). The APA is steeply income adjusted and paid to older adults aged 60 or over. Each level of dependency – according to the *autonomie gérontologie groupes iso-ressources* (AGGIR) grid – gives access to a maximum amount, which is then adjusted according to the recipient’s needs and level of income (see details in Table A.6). AGGIR grid distinguishes six levels of dependency, from *groupe iso-resource* (GIR) 1 (highest) to GIR 6 (lowest). Of the 6 GIR levels, four are entitled to financial help from the APA [24].

In contrast to Austria or Germany, for those cared for at home, the allowance is paid to finance a specific care plan elaborated by a multidisciplinary team (healthcare and social care professionals from the regional départements) after an assessment of needs. The program aims to support professional home care as well as facilitate the employment of family caregivers [25, 34]. Informal care arrangements are assumed to be involved in both care planning and service delivery and may include one or more family members (excluding the spouse) [35]. The benefit is typically paid directly to the beneficiary, and its use is monitored [24, 35].

Older individuals may receive other disability-related benefits from sources such as the p*restation de compensation du handicap* (PCH) [36–38]. Introduced in 2006 to better meet the complex needs of individuals with limitations in their daily activities, irrespective of age, the PCH is primarily dedicated to people whose disability already occurred before the age of 60 and is also allocated and financed by local authorities. While the APA is intended to support a pre-defined care mix, the PCH offers more flexibility, also allowing beneficiaries to use the funds for both formal and informal care. Individuals aged 60 or older who are eligible for both PCH and APA, however, must choose one benefit [36, 37].

While in 2015 the numbers of CFC users in France were comparable to those in Germany (5.5% in France), usage in France declined to 3% among older adults in need by 2021 (Fig. 1 and Table A.4).

## Materials and methods

### Data

This study uses data from the Survey of Health, Ageing and Retirement in Europe (SHARE), which is a representative survey of community-dwelling older adults aged 50 and above. SHARE collects comprehensive individual-level information on various health, (socio)economic and environmental indicators, including education, housing, social and financial support, and family structure. With the first wave conducted in 2004 and the most recent wave in 2021, new waves are published every two years [9]. As earlier waves lack information on key variables reflecting informal and formal personal care or CFC benefit receipt, data from the most recent waves – 6, 7, 8, and 9 – are used, spanning the years 2015 to 2021 [39–42]. The geographical scope of the analytical sample was limited to four European countries — Austria, Belgium, France, and Germany — for which data were available across all waves (see Table A.1). The analytical sample, restricted to individuals more likely to have care needs (defined by having at least one limitation with either IADL or ADL), leaves us with 10,283 observations, 1,909 from Austria, 3,661 from Belgium, 2,345 from France, and 2,368 from Germany (see Table 1 and for further details, Table A.3). Notably, the data is largely (repeated) cross-sectional, with only 596 observations from individuals observed in all four waves, and 5,834 observations from individuals observed at least twice.

## Outcome variables

### Informal and formal care use

Informal care is captured through a binary variable reflecting whether an individual has received unpaid personal care from informal resources. SHARE asks individuals whether family members, friends or neighbours — either from inside or outside their household — have regularly assisted them with personal care tasks, such as washing, getting out of bed, or dressing, during the past 12 months [9]. Similarly, formal care use is reported if a person has received regular help from any professional or paid personal care services. The reference category denotes that no informal (or, conversely, no formal) care use is reported, including no care use. Despite the primary aim to quantify the aggregate impact on informal and formal care use, respectively, results on combinations of informal and formal care use— referred to as *mixed care* — are provided in the Appendix to complement and provide further insights into our main results.

### Explanatory variables

#### CFC receipt

To capture whether an individual has received CFC benefits, individuals are asked whether they have gained income from public resources intended to meet LTC needs. In Austria, the SHARE questionnaire targets the publicly provided universal cash benefit. In Belgium, due to the fragmented system, the question more broadly covers any public cash benefits intended to cover LTC needs. Similarly, in France, individuals are asked about disability benefits related to LTC needs. Benefits received may not exclusively limit to payments from the national-wide program APA, intended for older people experiencing significant loss of autonomy or financial difficulties due to age-related conditions or disability, but include potential income from other regional and national-level programs of disability-related benefits intended to assist individuals with limitations in activities of daily living and help them manage the financial burden associated (e.g., the PCH). In Germany, the question refers to financial benefits received through the public LTCI scheme [9].

#### Control variables

The present study accounts for several individual-level factors known to influence the composition of the care mix. These include predisposing characteristics such as age, the severity of care need (IADL and ADL), and gender [43]. Among enabling factors, the presence of social resources (spouse or children), financial and material resources (wealth), and human resources (education) are considered [43, 44].

Following Courbage et al. [17], I estimate the model including controls for the use of formal and informal care, respectively. This allows to more precisely examine whether financial benefits determine care decisions by incentivizing or disincentivizing informal care — or conversely, formal care. To be able to capture variation across countries and waves, dummy variables are included for both.

### Instrumental variable

To address potential endogeneity, *granting generosity* (see further estimation details outlined below) was used as an instrument for *CFC receipt*. Granting generosity aims to reflect cross-wave, cross-national, and cross-regional variations in access to benefits when having LTC need. The regional units used reflect the existing federal states in Austria and Germany, while they correspond to provinces and administrative regions in Belgium and France (a full list of all regional units can be found in Table A.5). Specifically, for each individual within each wave, country, and region, the granting generosity is measured as the unique ratio of the number of older adults receiving benefits (excluding the individual in question) to the total number of older adults having at least 1 limitation with IADL or ADL.

### Estimation strategy

This analysis examines the relationship between care utilization (e.g., informal care) and the receipt of CFC benefits using a generalized linear model (GLM) with a logit link function.

1 $$\:log\left(\frac{P\:\left({Y}_{it}=1\right)}{1-P\:\left({Y}_{it}=1\right)}\right)=\:{\beta\:}_{0}+\:{{\beta\:}_{1}{CFC}_{it}+\beta\:}_{2}{X}_{it}+{\gamma\:}_{c}+{\delta\:}_{t}+{ϵ}_{it}$$

The GLM framework allows to model the binary nature of care utilization while controlling for relevant covariates $\:{X}_{it}$ [45]. $\:{Y}_{it}$ represents the outcome variable for individual *i* at wave *t*, where $\:{Y}_{it}=1$ if informal care (or, respectively, formal care) is received, and $\:{Y}_{it}=0$ if otherwise. $\:{CFC}_{it}$ indicates whether an individual has received cash benefits to meet LTC-related needs. $\:{\gamma\:}_{c}$ and $\:{\delta\:}_{t}$ represent country and wave fixed effects (FE), respectively. Standard errors are clustered at the individual level to account for any potential within-individual correlation over time.

In a second step, the analysis extends this framework by incorporating an instrumental variable approach (IV-GLM). Across countries, benefit schemes are organized through a needs-based approach, that is, CFC is eligible for those in need of personal care. While eligibility thresholds vary, they are often tied to IADL and/or ADL [17]. While already accounting for this by restricting the sample to those individuals more likely to have care needs [46], a simple GLM may suffer from potential endogeneity. Previous studies using administrative data have employed examiner allowance rates or leniency to study the effect of endogenous benefit receipt on labour market outcomes [47, 48] or health outcomes such as hospital admission, nursing home admission and overall mortality rates [49–51]. As direct measures on allowance rates are unavailable, we leverage the plausibly exogenous variation in generosity of granting CFC benefits (*granting generosity*) across waves, countries, and regions. In theory, more generous CFC schemes increase the likelihood of individuals qualifying for CFC benefits. That is, a large part of the variation in granting generosity comes from policy variations across waves and countries. Naturally, variations across regions may persist in more fragmented systems but also extend to countries with universal CFC schemes reflecting varying applications of eligibility criteria, such as differences in the average leniency of examiners across regions [23].

In the first stage, I model the likely endogenous variable $\:{CFC}_{it}$ (equaling 1 if benefits are received) using $\:{granting\:generosity}_{it}$ as an instrument:

2 $$\:log\left(\frac{P\:\left({CFC}_{it}=1\right)}{1-P\:\left({CFC}_{it}=1\right)}\right)=\:{\alpha\:}_{0}+\:{\alpha\:}_{1}{granting\:generosity}_{it}+{\alpha\:}_{2}{X}_{it}+{ϵ}_{it}$$

As stated, for each individual within each wave, country, and region, the $\:{granting\:generosity}_{it}$ measures a unique ratio relating the number of older adults receiving benefits – excluding the individual in question – to the number of older adults with personal care needs. In the first stage, I include $\:{X}_{it}$, a vector of individual-level control variables. As the instrument reflects cross-country and cross-wave differences, country and wave FE were omitted from the first stage to avoid overfitting (e.g., due to multicollinearity) and to retain some of the variability in the instrument. For sensitivity purposes, results including both country *and* wave FE in the first stage are provided in the Appendix.

In the second stage, I model the probability of receiving informal care ($\:{Y}_{it}=1)\:$using the predictors of the first stage. As stated, to adjust for the endogeneity of $\:{CFC}_{it}$, a control function approach, which includes the residuals from the first-stage regression (i.e., the part not explained by the instruments or other controls) as an additional explanatory variable in the second stage, is employed. This method explicitly accounts for the uncertainty and potential bias introduced during the first-stage estimation, ensuring more accurate standard errors and valid confidence intervals [52, 53].

3 $$\:log\left(\frac{P\:\left({Y}_{it}=1\right)}{1-P\:\left({Y}_{it}=1\right)}\right)=\:{\beta\:}_{0}+\:{\beta\:}_{1}{\widehat{CFC}}_{it}+{\beta\:}_{2}{X}_{it}+{\gamma\:}_{c}+{\delta\:}_{t}+{ϵ}_{it}$$

The variables and subscripts are the same as those defined in Eq. (2) and our coefficient of interest $\:{\beta\:}_{1}$ measures the impact of receiving CFC benefits on the probability of using informal care. Analogous to Eq. (3), where $\:{Y}_{it}$​ represents informal care use, I perform the analysis using formal care as the dependent variable. To be able to explore heterogeneities across countries, interaction terms between CFC and country were introduced in a subsequent step.

#### Instrument validity and further sensitivity analyses

To further validate the approach and ensure that granting generosity meets the required criteria of relevance and exogeneity, a series of tests were conducted, including a thorough investigation of first-stage results using different model specifications. To assess exogeneity, a falsification test, regressing (in)formal care use on granting generosity among those not receiving CFC benefits, was performed. To ensure monotonicity assumption holds, it is shown that granting generosity consistently increases the likelihood of receiving CFC across different subpopulations, with no reverse effects.

More generally, to address potential issues with sample size in smaller regions, sensitivity of the chosen instrument was evaluated by defining granting generosity using broader regional NUTS-1 classifications instead of NUTS-2 level data, which provides a less granular geographical breakdown. Additional sensitivity tests were performed by (i) excluding Belgium due to a low number of CFC recipients and (ii) restricting the highly unbalanced sample to individuals observed at least twice, thus creating a more balanced sample. Further sensitivity tests include using ADL solely instead of both IADL and ADL as sample restriction indicator for care need.

## Results

Although informal care is the most frequent type of care, even among those not receiving CFC, first descriptive statistics in Table 1 show that reliance on family-based care (i.e., informal care) is more common among those receiving CFC. While, overall, 23% of older adults with care needs (i.e., having at least 1 IADL or ADL) receive informal care, more than half of those reporting to receive CFC benefits also receive informal care (50.5%). It is important to note, however, that care utilization is higher among CFC recipients (and, conversely, no care use is lower), and that also the share of respondents using formal care is higher (34.9% compared to 15.1% overall). Moreover, CFC recipients, on average, report more severe limitations in IADL and ADL, are more likely to be female, and less likely to have a spouse, children, or higher levels of education or wealth (Table 1).

Despite existing similarities, country-specific differences become evident. Except for France, where a negative trend in uptake of CFC can be observed, Fig. 1 (see Sect. 2) reveals a sizeable expansion in CFC usage in Austria, Belgium, and Germany throughout the period investigated. Yet, also within the countries that share a positive trend, there are differences in both the rate of expansion and the average prevalence across waves (Table 1 and Table A4). While benefit receipt is comparatively lower in France (4.1%) and Belgium (1.6%), the German-speaking countries, including Austria (28.6%) and Germany (7.2%), rely most heavily on CFC. Correspondingly, both informal and formal care services use remains higher in Austria and Germany, although their relative importance is different, with a slightly higher average prevalence of formal (and mixed) care use and a slightly lower average prevalence of informal care use in Germany than in Austria (Table A.1). Yet, across waves, a consistent negative trend in the use of informal care over time is observed across countries (Table A.2). As for formal care use, notable increases in its use since 2015 in Belgium (from 13.5% to 16.1% in 2021) and comparably more modest increases in France (from 11.2% to 12.3% in 2021; see Table A.2) can be reported.

**Table 1:** Descriptive sample statistics by CFC benefit receipt and overall

	CFC non-receipt (*N* = 9,418)	CFC receipt (*N* = 865)	Overall (*N* = 10,283)
informal care	1,933 (20.5%)	437 (50.5%)	2,370 (23.0%)
formal care	1,254 (13.3%)	302 (34.9%)	1,556 (15.1%)
*mixed care*	*641 (6.8%)*	*195 (22.5%)*	*836 (8.1%)*
*no care*	*6*,*872 (73.0%)*	*321 (37.1%)*	*7*,*193 (70.0%)*
age	75.1 (10.8)	79.4 (10.5)	75.5 (10.9)
number of limitations with…			
*IADL*	2.21 (2.28)	4.58 (2.82)	2.41 (2.42)
*ADL*	1.07 (1.43)	2.22 (2.02)	1.16 (1.52)
gender: female *(ref. male)*	5,871 (62.3%)	573 (66.2%)	6,444 (62.7%)
spouse *(ref. no parnter)*	5,303 (56.3%)	378 (43.7%)	5,681 (55.2%)
children *(ref. no children)*	8,226 (87.3%)	750 (86.7%)	8,976 (87.3%)
wealth	252,000 (335,000)	132,000 (287,000)	242,000 (333,000)
education	11.1 (3.91)	9.60 (4.53)	11.0 (3.98)
country			
*Austria*	1,367 (14.5%)	542 (62.7%)	1,909 (18.6%)
*Belgium*	3,604 (38.3%)	57 (6.6%)	3,661 (35.6%)
*France*	2,249 (23.9%)	96 (11.1%)	2,345 (22.8%)
*Germany*	2,198 (23.3%)	170 (19.7%)	2,368 (23.0%)

For age, IADL, ADL, wealth, and education, the numbers indicated in brackets represent the standard deviation

### Estimation results

Table 2 presents regression results with both informal and formal care as dependent variables, using two main models: (i) a GLM, and (ii) an IV-GLM exploiting the exogenous variation in accessing these CFC benefits across waves, countries, and regions (*granting generosity*). Considering a range of factors associated with care use, such as the severity of care needs, the presence of social resources (e.g., a spouse or children), and the education level, the results in Table 2 consistently suggest a positive impact of CFC receipt on informal care use. Both the GLM and the IV-GLM results (columns 1–4) show a strong, positive, and statistically significant association between CFC receipt and informal care use (*p* < 0,01). Yet, the coefficient becomes even larger when accounting for heterogeneity in the granting generosity as instrument for receiving CFC benefits. That is, the IV-GLM in column 3 predicts that individuals receiving CFC are about 3 times more likely to use informal care *(exp(1.097) = 2.996*), assuming other variables in the model are held constant. Further, geographical differences become evident, with the positive impact on family-based informal care being significantly more pronounced in Austria and Germany (column 4). The opposite appears to be true for Belgium. However, due to the limited number of observations of individuals receiving CFC benefits in Belgium, coefficients must be interpreted with greater caution.

While in both the GLM and IV-GLM the direction of the effect remains consistent for informal care, this is not the case for formal care (columns 5–8). Albeit results from a simple GLM in column 5 show a positive, though – in comparison to informal care – smaller, impact of CFC receipt on formal care use, the positive coefficient disappears entirely and even reverses when using an IV-based approach. Despite the main results indicating a negative relationship between CFC receipt and formal care use (column 7), notable cross-country differences emerge: the negative impact on formal care use is particularly pronounced in Austria, while it is not evident in Germany, the only country in which CFC has a positive – yet only weakly significant (*p* < 0,10) – impact on the use of formal care services (column 8). Notably, informal and formal care use are not mutually exclusive and may include mixed as well as exclusive forms of care. Additional findings exclusively considering mixed care – a combination of informal and formal care use – complement these results and reveal that the contrasting findings for Germany are related to significant increases in mixed care use (*p* < 0,01; Table B.1). Moreover, the results in Table B.1 indicate that CFC receipt significantly reduced ‘no care’ use in Germany (*p* < 0,01). Although sample size is further reduced, the results are robust to varying restrictions to the analytical sample, such as those having at least 1 ADL^1^, as well as the use of a more balanced sample that solely includes respondents who were observed at least twice in the sample (Table B. 2). Notably, the included wave FE show no systematic change in formal care use over time but indicate a significant decline in informal care use from wave 8 onward, partially coinciding with the COVID-19 pandemic (see also Table A.2). SHARE wave 8 (2019–2020) began just before the pandemic and was partially disrupted, while wave 9 (2021–2022) covers its later stages [41, 42]. Yet, the wave FE capture all systematic differences between waves; thus, other temporal factors – including broader trends in LTC – may also contribute. By wave 9, most direct pandemic restrictions had been lifted, suggesting that immediate effects may have subsided, while indirect or residual effects – such as altered caregiving patterns or delayed (in)formal care – could persist [54–58].

**Table 2:** Regression results on informal and formal care use (GLM vs. IV-GLM comparisons)

	**Dependent variable:**							
	**Informal care**				**Formal care**			
	**GLM**		**IV-GLM**		GLM		**IV-GLM**	
	**(1)**	**(2)**	**(3)**	**(4)**	**(5)**	**(6)**	**(7)**	**(8)**
age	-0.002	-0.002	-0.003	-0.000	0.037^***^	0.037^***^	0.040^***^	0.037^***^
	(0.003)	(0.003)	(0.003)	(0.003)	(0.004)	(0.004)	(0.004)	(0.004)
iadl	0.148^***^	0.151^***^	0.148^***^	0.151^***^	0.169^***^	0.171^***^	0.171^***^	0.171^***^
	(0.015)	(0.015)	(0.015)	(0.015)	(0.016)	(0.016)	(0.016)	(0.016)
adl	0.323^***^	0.325^***^	0.323^***^	0.325^***^	0.226^***^	0.227^***^	0.224^***^	0.224^***^
	(0.022)	(0.022)	(0.022)	(0.022)	(0.024)	(0.024)	(0.024)	(0.024)
female (ref. male)	-0.055	-0.052	-0.056	-0.056	0.152^**^	0.154^**^	0.156^**^	0.159^**^
	(0.057)	(0.057)	(0.057)	(0.057)	(0.071)	(0.071)	(0.071)	(0.071)
children (ref. none)	0.369^***^	0.361^***^	0.364^***^	0.369^***^	-0.092	-0.097	-0.083	-0.083
	(0.094)	(0.094)	(0.094)	(0.094)	(0.098)	(0.098)	(0.098)	(0.097)
spouse (ref. none)	0.987^***^	0.991^***^	0.989^***^	0.999^***^	-0.571^***^	-0.573^***^	-0.577^***^	-0.578^***^
	(0.068)	(0.068)	(0.068)	(0.068)	(0.079)	(0.079)	(0.079)	(0.079)
wealth quintiles (ref: 1st quintile)								
*2nd quintile*	0.046	0.054	0.065	0.024	0.074	0.080	-0.018	0.063
	(0.077)	(0.077)	(0.079)	(0.084)	(0.088)	(0.088)	(0.092)	(0.099)
*3rd quintile*	0.051	0.059	0.075	0.022	0.154	0.155	0.083	0.142
	(0.083)	(0.083)	(0.085)	(0.092)	(0.098)	(0.098)	(0.101)	(0.113)
*4th quintile*	-0.010	-0.010	0.015	-0.042	0.071	0.067	-0.004	0.062
	(0.088)	(0.089)	(0.091)	(0.093)	(0.109)	(0.110)	(0.112)	(0.125)
*5th quintile*	0.016	0.018	0.045	-0.020	-0.040	-0.044	-0.130	-0.054
	(0.098)	(0.099)	(0.101)	(0.111)	(0.129)	(0.130)	(0.133)	(0.235)
education (years)	-0.018^**^	-0.019^***^	-0.018^**^	-0.018^**^	-0.010	-0.011	-0.011	-0.011
	(0.007)	(0.007)	(0.007)	(0.007)	(0.009)	(0.009)	(0.009)	(0.009)
formal care	1.150^***^	1.142^***^	1.154^***^	1.153^***^				
	(0.075)	(0.076)	(0.075)	(0.076)				
informal care					1.239^***^	1.229^***^	1.245^***^	1.244^***^
					(0.073)	(0.074)	(0.073)	(0.073)
CFC receipt (ref. non-receipt)	0.644^***^	0.602^***^	1.097^***^	0.812^**^	0.495^***^	0.338^**^	-0.911^*^	-1.028^**^
	(0.100)	(0.137)	(0.393)	(0.406)	(0.117)	(0.154)	(0.469)	(0.482)
country (ref. Austria)								
*Belgium*	-0.394^***^	-0.372^***^	-0.299^***^	-0.011	0.623^***^	0.557^***^	0.309^**^	0.131
	(0.085)	(0.089)	(0.116)	(0.158)	(0.111)	(0.116)	(0.145)	(0.21)
*France*	-0.356^***^	-0.390^***^	-0.264^**^	-0.365^**^	0.132	0.068	-0.172	-0.238
	(0.090)	(0.096)	(0.117)	(0.161)	(0.118)	(0.126)	(0.149)	(0.206)
*Germany*	-0.078	-0.118	0.008	-0.213	0.717^***^	0.584^***^	0.435^***^	0.222
	(0.087)	(0.094)	(0.113)	(0.147)	(0.112)	(0.122)	(0.141)	(0.180)
CFC × *Belgium*		-1.368^***^		-9.074^***^		-0.210		3.218
		(0.351)		(3.001)		(0.466)		(3.908)
CFC × *France*		0.430		1.045		-0.045		0.660
		(0.283)		(2.245)		(0.408)		(2.606)
CFC × *Germany*		0.398		3.058^**^		0.609^**^		2.661^*^
		(0.244)		(1.390)		(0.257)		(1.484)
Observations	10,283	10,283	10,283	10,283	10,283	10,283	10,283	10,283
Wave FE	Yes	Yes	Yes	Yes	Yes	Yes	Yes	Yes

For consistency between GLM and IV-GLM, coefficients displayed as log-odds; Standard errors in brackets ^*^p < 0.1; ^**^p < 0.05; ^***^p < 0.01

### First-stage results and instrument validity

Table 3 displays first-stage results, adding control variables and fixed effects in a stepwise manner (columns 1–2). Notably, the instrument is strongly significant across all model specifications. A sizeable drop in coefficient size from column (2) (as used in the main model in Table 2) to column (3), however, shows that a large part of the variation in the instrument comes from cross-wave, and more so, cross-country heterogeneity in access to these benefits. Still, the instrument remains sufficiently strong when accounting for country and wave FE in the first stage, indicating that significant regional variation in the instrument, as shown in column 3, persists. Sensitivity tests based on Model 2 (column 3 in Table B.3) also yield consistent results, further supporting the validity of the IV-GLM results. Including FE in the first stage, particularly country FE, renders, however, some of the interaction effects with country obsolete, while the main effects become stronger.

**Table 3:** First-stage regression results on CFC receipt

	**Dependent variable: CFC receipt**		
	**Model 1**		**Model 2**
	**(1)**	**(2)**	**(3)**
granting generosity	7.873^***^	7.627^***^	3.270^***^
	(0.266)	(0.280)	(0.509)
Covariates	No	Yes	Yes
Wave FE	No	No	Yes
Country FE	No	No	Yes
Observations	10,283	10,283	10,283

Coefficients as log-odds; Standard errors in brackets ^*^p < 0.1; ^**^p < 0.05; ^***^p < 0.01

Additional tests were performed supporting that the instrument (*granting generosity)* satisfies the *relevance* criterion (it is strongly correlated with receiving CFC) and the *exclusion* restriction (it affects informal and formal care use only through CFC receipt). In a first step, instrument relevance is supported by comparing the t-statistic to a critical value (e.g., 1.96 for a 95% confidence interval). Approximating instrument strength based on a linear version of the model (as the IV-GLM framework does not naturally allow for an F-test for weak instruments) further supports that the instrument is sufficiently strong, with F-statistics of 90.1 for informal care and 86.1 for formal care [52]. While weak instrument diagnostics are less formalized for nonlinear models, the results provide additional confidence in the overall validity of the instrument. To support exogeneity, a falsification test was performed regressing informal and formal care use on granting generosity within the subsample of individuals not receiving benefits (Table A.6). This analysis demonstrates that the instrument affects the outcome variable only indirectly through the endogenous variable, CFC receipt. The fact that the falsification test holds suggests that granting generosity is capturing the intended variation in benefit receipt, without being confounded by long-term trends or other time-varying factors that may be less controlled for. Finally, regressing the endogenous variable (CFC receipt) on granting generosity across different subgroups (e.g. age or gender) confirms that the monotonicity assumption holds, further supporting the general validity of the chosen IV approach (for reference, see Table C.7 ) [59].

Results using an alternative measurement of the granting generosity considering broader territorial units based on NUTS-1 regional information are shown in Table B.4. Additionally, I provide results excluding Belgium due to its comparatively low number of CFC recipients and resulting potential to bias results due to sample size issues (Table B.5). Both results are provided in the Appendix and remain consistent.

Finally, during the study period, regulatory changes to CFC schemes, eligibility thresholds, and the broader LTC system took place. For instance, in Belgium, the APA fell under the jurisdiction of the communities, while in Austria, a reform affecting residential care came into force in 2018 (Federal Law Gazette no. 125/2017), prohibiting the state from reclaiming the assets of care recipients or their relatives to finance institutional care. While regulations affecting eligibility are captured by our instrumental variable — since reforms that make access to benefits more or less restrictive directly affect granting generosity — we also account for any other time-varying (policy) change by estimating models with country-wave interactions included (see Table B.9). Although the country FE included in our main model absorb cross-country differences (e.g., in allowance amounts), they do not account for within-country or temporal variation. The coefficients for CFC receipt remain largely unchanged when country–wave interactions are included, providing additional confidence that the associations with informal and formal care use are not driven by within-country policy reforms (e.g., changes in benefit amounts) nor shifts in eligibility rules over time. It is noteworthy that although the focus of this study lies on benefit receipt rather than the intensity of the amount, additional information on CFC amounts would be highly informative. Despite the lack of reliable individual-level data on benefit amounts, analyses that include average country–wave-level benefit amounts (as an additional control) yield consistent results (Table B.8), suggesting that the main effect reflects the impact of receiving a benefit rather than its monetary size.

## Discussion and conclusion

This study addresses crucial gaps in research regarding the overall effectiveness of CFC benefits, particularly as evidence on their impact on both informal and formal care use – especially in Continental European countries – remains limited. Widely regarded as a more cost-effective approach to finance LTC in the context of demographic ageing and changing family and household structures [6, 60], CFC remains particularly prevalent in countries historically taking a family-based approach towards LTC. Using SHARE data from 2015 to 2021 across four of these countries — Austria, Belgium, France, and Germany — this study provides novel evidence on the aggregate impact of these schemes, applying generalized linear models (GLMs) and exploiting the exogenous variation in the granting of CFC benefits. Findings reveal that providing publicly funded cash benefits likely affects both informal *and* formal care use. Yet, despite existing similarities, effects are not homogenous across countries, suggesting that the specific design and application of the CFC scheme in place – particularly differences in access to these benefits – play a crucial role in whether (in)formal use is encouraged.

In line with previous evidence for Italy or Spain [7, 17], results from a GLM consistently point towards CFC fostering the use of family-based informal care across Continental European countries, thus not leading to a ‘crowding-out’ of the family as proposed by ‘substitution theory’ and instead being consistent with ‘encouragement theory’ stating that use of informal care rises as opportunity costs arising from paid employment in the labour market likely become lower [12]. This generally aligns with the notion that the countries investigated largely continue to promote the role of the family, which remains consistent with the basic principles of conservative-corporatist welfare state models [8]. However, differences between countries and their CFC schemes become apparent and were shown to play a decisive role in promoting a specific type of care. As for informal care use, the positive impact is particularly pronounced among Austrian, but also German older adults, where coverage rates are comparatively high and use of CFC remains highly unregulated [60, 61]. Albeit Austria stands out for its broad coverage (with 28.6% of older adults in need receiving CFC, compared to 7.2% in Germany, 4.1% in France, and 1.6% in Belgium), the distribution of recipients across the benefit levels – and, consequently, the exact payments allocated – likely vary [25, 62]. For instance, as for Austria, previous literature shows that nearly half of the recipients fall into the two lowest benefit levels [23]. Additional within and between-country variation occurs in the amounts granted within each care allowance level (see Table A.6.). Albeit there are no formal restrictions on its use, CFC has been conceived as a means to support family caregivers in their caring activities [16, 61]. While it is argued that CFC marked a clear shift from unsupported towards supported *familialism*, the French and, notably also the Belgian reforms did not follow this same trajectory – or at least not in the same way [63]. While the impact on informal care in France is also positive - albeit less pronounced - supporting families was not the only policy objective of the main CFC scheme introduced in 2002 – the APA. Although informal care (excluding care provided by a spouse) may be included in the pre-defined care plan, it has been argued that CFC was also introduced to reduce unemployment by expanding the home care sector [16].

Notably, differences emerge as to whether formal care use is affected. Identification from plausible exogenous variation in granting of CFC benefits reveals that, on average, CFC discourages formal care use. Although the main effect remains only weakly significant (*p* < 0.10), it becomes particularly pronounced in Austria (*p* < 0.05), while in Germany it reverses and even becomes positive. That is, cash payments – received through Germany’s statutory LTCI – significantly encourage informal *and* formal care, particularly through higher mixed care use. Notably, the German CFC scheme, known to foster shared responsibility between informal and formal care arrangements, was found to effectively increase care use overall and hence reduce potential unmet needs. While SHARE does not allow differentiation between formal public and formal private care, additional statistics based on waves 6 and 7 (*no more recent information available*, see Table A.7) reveal that Germany has the lowest share of individuals using out-of-pocket (OOP) payments for home care. In contrast, Austria exhibits relatively high share of OOP users, accompanied by comparatively higher mean OOP expenditures, which may act as an additional barrier to accessing formal care. It should be noted that the available information on OOP only captures expenses paid directly by the individual without reimbursement; private payments covered by insurance are not included. Therefore, these data serve as a proxy rather than a complete measure of privately funded formal care. Further research examining the role of public versus private formal care, as well as the nature and type of provision (by professional caregiver or unqualified domestic workers), is needed to provide additional insights.

Given the increasing pressure on the availability of (informal) caregivers and likely substantial external costs of informal caregiving more generally [64], these insights are crucial for policy-making focused on alleviating pressure on informal caregivers — particularly women — while addressing unmet needs amidst rising care demand. CFC schemes, as implemented in Germany and Austria, can enhance access to LTC by granting recipients flexibility and autonomy in organizing care, increasing choice for families, and supporting aging in place. However, evidence indicates that without careful regulation, such schemes may discourage the use of formal LTC services and shift care responsibilities onto informal caregivers, potentially undermining their employment and health outcomes [11, 65, 66]. Against the backdrop of historically high levels of family care use, demographic and societal changes (rising female labor force participation, evolving cultural norms around gender roles, workforce contraction, and population aging) are set to sharply constrain the future availability of informal caregivers. Policymakers may therefore balance support for family carers with measures that promote the use of formal LTC services. Policy options include strengthening in-kind benefits, investing in accessible formal care infrastructure, and designing incentives that encourage the use of professional care, as exemplified by the German model. While countries with similar welfare regimes encounter common pressures, the optimal balance between formal and informal care support should be tailored to each nation’s demographic, labor, and social context, offering transferable lessons for addressing growing unmet care needs.

Despite existing similarities, variations in political context, timing, and the specific regulation of benefit schemes may have led to differing visions of care and care work, resulting in distinct LTC configurations. Overall, in contrast to Southern European countries with ‘strong family ties’ and the Northern European countries with ‘weak family ties’, Continental European countries (particularly Austria, France, and Germany) have moved – albeit to varying degrees – from an unsupported family-based approach to elderly care to a mixed model combining public measures (i.e., primarily cash benefits) and family-based care (i.e., informal care) [1, 67]. That is, despite recent declines in CFC use in France, overall, in Austria, France, and Germany, CFC is regarded as the main pillar of LTC policies. In contrast, the situation in Belgium is arguably different, with CFC playing a comparatively minor – though also increasing – role, as data show. Although recent efforts have focused on deinstitutionalizing LTC, reports by the European Commission reveal that Belgium’s LTC system today may be closer to the Northern European countries, relying not only cash benefits but also substantial in-kind benefits and care provided outside the home taking a larger role (e.g., in institutions) [24].

The traditional role of the family in conservative-corporatist welfare states, coupled with the growing reliance on cash – rather than in-kind – benefits in many European countries, has raised questions about the potential impact on the care mix. While this study provides first insights into the role of CFC and its potential to encourage family-based informal, while largely discouraging formal care use (except for Germany), there are some limitations that merit attention. First, a potential attrition in the data due to mortality or institutionalization (e.g., nursing home entry), which is particularly common in longitudinal studies of older adults, might affect the results [49, 68]. In this study, this concern is, however, mitigated for two reasons. First, the IV strategy exploits variation at the cross-country, cross-wave, and cross-regional levels, rather than relying on individual-level variation. This reduces the risk that selective dropout directly biases our estimates. Second, despite attrition, SHARE data remains representative of the older population living in the community in each wave [9]. Since this study uses largely repeated cross-sections (with 43% of the participants in the sample observed only once, and only 6% of the respondents observed in all four waves) rather than a strict panel approach, the results remain generalizable to the broader population, which is also the intended aim of this study.

Further, albeit the chosen approach mitigates major concerns about bias stemming from unobserved variations in access to these CFC benefits, it remains limited in fully eliminating all sources of potential endogeneity. To identify trends in informal and formal care use, as well as the role of CFC, unobserved heterogeneity at the country and wave level was considered. Despite several robustness tests supporting that results are not driven by within-individual variation (e.g., by using a more balanced sample), due to the highly unbalanced nature of the data, accounting for individual-level heterogeneity is limited. As country and wave FE absorb most of the clustering effects in the data, individual FE could not be estimated, and additional attempts to include random intercepts for individuals, despite revealing consistent results, did not converge due to insufficient within-individual variability. Although this study focuses on the aggregate impact of CFC, emphasizing country-level differences rather than individual-level variations, future research could build upon this using panel data and explore within-individual heterogeneity more specifically.

Finally, while the chosen model and IV strategy account for the likelihood of receiving a cash benefit, it does not directly capture the actual monetary value of the benefit. Despite additional tests controlling for the average amount and country–wave interactions (see Table B.8 and B.9), which help account for time-varying policy changes and cross-country variation in benefit generosity, our approach cannot fully separate the effect of receiving a benefit from the effect of its monetary amount due to missing individual-level data. Building upon these findings using administrative data could thus be a fruitful avenue for research, as it allows further details of the CFC scheme to be considered, including the distribution and specific amount of the benefit received.

In conclusion, this study analysed the impact of CFC receipt on care use in four European countries (Austria, Belgium, France, and Germany) using cross-national data from SHARE. Examining changes in use at the extensive margin (i.e., whether informal or formal care is utilized), the findings indicate that CFC fostered family-based informal care over the period studied, though the exact magnitude varied across countries. In contrast, CFC did not lead to comparable increases in formal care use, except in Germany, where both informal *and* formal care — primarily through higher mixed care use — was encouraged. While further research is needed exploring the intensive margin (i.e., the amount of informal and formal care provided), this study marks an important first step in assessing the effectiveness of CFC schemes – especially in light of lacking data and evidence regarding the impact on home-based LTC. The findings hold important policy implications for the organization of LTC and healthcare systems, especially as efforts to reduce overall costs may be balanced with the need to alleviate the (informal) caregiver burden while ensuring both quantity and quality of care.

## Supplementary Information

Below is the link to the electronic supplementary material.

Supplementary Material 1

## Footnotes

1 Results can be provided upon request.
