# Supplementary material for: Cash-for-care and its impact on older adults’ informal and formal long-term care use: evidence from continental European countries
Source: Eur J Health Econ. 2026 Mar 4;27(7):1525–38. doi: 10.1007/s10198-026-01901-y (PMC13619754; doi:10.1007/s10198-026-01901-y)
Supplement: Supplementary file 1 [file 10198_2026_1901_MOESM1_ESM.docx]

**Appendix**

**Cash-for-Care and Its Impact on Older Adults’ Informal and Formal Long-Term Care Use: Evidence from Continental European Countries**

**Appendix A**

Table A.1: Summary statistics by country

|  | **Austria *(N=1909)*** | **Belgium *(N=3661)*** | **France *(N=2345)*** | **Germany *(N=2368)*** |
| --- | --- | --- | --- | --- |
| **Informal care** | 567 (29.7) | 713 (19.5%) | 450 (19.2%) | 640 (27.0%) |
| **Formal care** | 309 (16.2%) | 555 (15.2%) | 280 (11.9%) | 417 (17.4%) |
| ***Mixed care*** | *196 (10.3%)* | *219 (6.0%)* | *136 (5.8%)* | *285 (12.0%)* |
| ***No care*** | *1,229 (64.4%)* | *2,612 (71.3%)* | *1,751 (74.7%)* | *1,601 (67.6%)* |
| **CFC receipt** (ref. non-receipt) | 542 (28.4%) | 57 (1.6%) | 96 (4.1%) | 170 (7.2%) |
| **Age**  Mean (SD) | 77.7 (9.62) | 74.6 (11.2) | 76.6 (11.2) | 73.8 (10.5) |
| **Number of limitations with…** |  |  |  |  |
| **IADL** | 2.87 (2.58) | 2.36 (2.36) | 2.21 (2.34) | 2.33 (2.40) |
| **ADL** | 1.13 (1.69) | 1.11 (1.42) | 1.13 (1.45) | 1.31 (1.59) |
| **Gender (**ref. male) | 1256 (65.8%) | 2354 (64.3%) | 1529 (65.2%) | 1305 (55.1%) |
| **Spouse** (ref. no parnter) | 939 (49.2%) | 2023 (55.3%) | 1226 (52.3%) | 1493 (63.0%) |
| **Children** (ref. no children) | 1655 (86.7%) | 3165 (86.5%) | 2082 (88.8%) | 2074 (87.6%) |
| **Wealth**  Mean (SD) | 140000 (278000) | 318000 (381000) | 253000 (289000) | 196000 (305000) |
| **Education** Mean (SD) | 8.69 (4.41) | 11.6 (3.67) | 10.8 (3.81) | 12.1 (3.45) |

Table A.2: Informal, formal, mixed and no care use by country and wave

| Country | Wave | Informal care (%) | Formal care (%) | Mixed care (%) | No care  (%) |
| --- | --- | --- | --- | --- | --- |
| ***Austria*** | 6 | 30.4 | 17.6 | 11.5 | 63.4 |
|  | 7 | 30.5 | 15.6 | 8.4 | 62.3 |
|  | 8 | 30.3 | 14.5 | 10.3 | 65.5 |
|  | 9 | 28.1 | 15.6 | 9.1 | 65.4 |
| ***Belgium*** | 6 | 20.6 | 13.5 | 6.1 | 72.0 |
|  | 7 | 21.1 | 16.8 | 5.8 | 67.9 |
|  | 8 | 19.3 | 16.7 | 5.5 | 69.5 |
|  | 9 | 17.1 | 16.1 | 6.2 | 73.0 |
| ***France*** | 6 | 19.7 | 11.2 | 6.4 | 75.5 |
|  | 7 | 20.7 | 10.7 | 4.7 | 73.2 |
|  | 8 | 19.1 | 13.3 | 5.8 | 73.4 |
|  | 9 | 17.2 | 12.3 | 5.3 | 75.7 |
| ***Germany*** | 6 | 27.8 | 21.1 | 16.1 | 67.2 |
|  | 7 | 29.9 | 24.6 | 16.2 | 61.7 |
|  | 8 | 25.2 | 17.0 | 11.4 | 69.2 |
|  | 9 | 27.1 | 12.7 | 7.7 | 68.0 |

Table A. 3: Sample statistic by country and wave

| **Country** | **Wave** | | | | **Total** |
| --- | --- | --- | --- | --- | --- |
|  | ***6*** | ***7*** | ***8*** | ***9*** |  |
| ***Austria*** | 777 | 154 | 406 | 572 | 1909 |
| ***Belgium*** | 1,557 | 483 | 581 | 1,040 | 3661 |
| ***France*** | 938 | 299 | 638 | 470 | 2345 |
| ***Germany*** | 769 | 167 | 630 | 802 | 2368 |
| ***Sum*** | 4041 | 1103 | 2255 | 2884 | 10283 |

**Instrumental variable** *(Descriptive statistics)*

Table A. 4: Summary statistics on instrumental variable by country and wave (granting generosity)

| Country | Wave | Granting generosity (%) | |
| --- | --- | --- | --- |
|  |  | *Mean* | *SD* |
| ***Austria*** | 6 | 24.2 | 4.2 |
|  | 7 | 36.4 | 16.4 |
|  | 8 | 29.8 | 11.1 |
|  | 9 | 30.9 | 9.0 |
|  |  | *28.4* | *9.7* |
| ***Belgium*** | 6 | 1.3 | 1.8 |
|  | 7 | 1.2 | 2.2 |
|  | 8 | 1.5 | 2.2 |
|  | 9 | 2.1 | 3.0 |
|  |  | *1.6* | *2.4* |
| ***France*** | 6 | 5.5 | 3.6 |
|  | 7 | 2.3 | 2.0 |
|  | 8 | 3.6 | 3.6 |
|  | 9 | 3.0 | 3.2 |
|  |  | *4.1* | *3.6* |
| ***Germany*** | 6 | 6.1 | 4.5 |
|  | 7 | 7.8 | 6.9 |
|  | 8 | 7.9 | 5.9 |
|  | 9 | 7.5 | 4.4 |
|  |  | *7.2* | *5.1* |

Table A.5: Number of observations per region (basis for instrumental variable)

| **Austria**  ***States*** *(Bundeslaender)* | | **Belgium**  ***Provinces*** *(+ Brussels)* | | **France**  ***Regions*** | | **Germany**  ***States***  *(Bundeslaender)* | |
| --- | --- | --- | --- | --- | --- | --- | --- |
| Burgenland | 157 | Brussels | 253 | Ile de France | 235 | Baden-Wuerttemberg | 229 |
| Niederoesterreich | 294 | Antwerpen | 637 | Champagne-Ardenne | 47 | Bayern | 358 |
| Wien | 431 | Limburg | 215 | Picardie | 52 | Berlin | 76 |
| Kaernten | 86 | Oost-Vlaanderen | 332 | Haute-Normandie | 42 | Brandenburg | 117 |
| Steiermark | 390 | Vlaams-Brabant | 254 | Centre | 105 | Bremen | 33 |
| Oberoesterreich | 334 | West-Vlaanderen | 445 | Basse-Normandie | 84 | Hamburg | 25 |
| Salzburg | 104 | Brabant Wallon | 169 | Bourgogne | 53 | Hessen | 186 |
| Tirol | 57 | Hainaut | 442 | Nord - Pas-de-Calais | 174 | Mecklenburg-Vorpommern | 53 |
| Vorarlberg | 56 | Liege | 613 | Lorraine | 117 | Niedersachsen | 243 |
|  |  | Luxembourg | 112 | Alsace | 55 | Nordrhein-Westfalen | 435 |
|  |  | Namur | 189 | Franche-Comte | 47 | Rheinland-Pfalz | 99 |
|  |  |  |  | Pays de la Loire | 189 | Saarland | 15 |
|  |  |  |  | Bretagne | 119 | Sachsen | 200 |
|  |  |  |  | Poitou-Charentes | 68 | Sachsen-Anhalt | 97 |
|  |  |  |  | Aquitaine | 186 | Schleswig-Holstein | 111 |
|  |  |  |  | Midi-Pyrenees | 111 | Thueringen | 91 |
|  |  |  |  | Limousin | 43 |  |  |
|  |  |  |  | Rhone-Alpes | 291 |  |  |
|  |  |  |  | Auvergne | 74 |  |  |
|  |  |  |  | Languedoc-Roussillon | 138 |  |  |
|  |  |  |  | Provence-Alpes-Cote d'Azur | 115 |  |  |
|  |  |  |  | Corsica | 0 |  |  |

Table A.6: Overview of main cash-for-care scheme in selected European countries

| **Country** | **Main cash-for-care scheme**  **for older adults** | **Introduction date** | **Benefit levels (per month)** | **Choice restrictions** | | |
| --- | --- | --- | --- | --- | --- | --- |
|  |  |  |  | ***Discretionary use*** | ***Free choice between cash and in-kind*** | ***Means-testing***^[1]^ |
| ***Austria*** | Bundespflegegeld | 1993 | 7 levels (2021):  €162.50 - €1745.10 | Yes | No | No |
| ***Belgium*** | Several cash-for care schemes *(responsibility lies within the Flemish-speaking, French-speaking, and German-speaking Communities*) | 1989 (major reforms in 2001 and 2014) | *Integration allowance (allocation d'intégration/integratietegemoetkoming): € 1,297.28 - € 11,243.20 (yearly amounts, 5 levels; 2021)*  *Allowance for assistance to the elderly (allocation pour l'aide aux personnes âgées/tegemoetkoming voor hulp aan bejaarden: yearly amounts – monthly payments; 2021):*  *€1,062.55 - €7,132.63 (5 levels)*  *In Flanders, the allowance has been integrated into the Vlaamse Sociale Bescherming (VSB):*   - *Care budget for severely dependent persons (zorgbudget voor zwaar zorgbehoevenden): fixed monthly amount of € 130.* - *Care budget for persons with disabilities (zorgbudget voor mensen met een handicap): fixed monthly amount of € 300.* - *Care budget for elderly persons requiring care (zorgbudget voor ouderen met een zorgnood): between €1,062,60 and €7,132.97 (2021)* | Yes | No | Yes *(partially)^[2]^* |
| ***France*** | Allocation personnalisée d’autonomie (APA)^[3]^ | 2002 | APA/4 levels (maxmum amounts, 2024^[4]^)  €746.54 (GIR 4) – €1,914.04 (GIR 1).  *Note: Dependency levels are classified according to Iso-resource groups (Groupes Iso Resources—GIR). Of the 6 IRG (GIR), four are entitled to financial help from the APA.* | No^[5]^ | No | Yes |
| ***Germany*** | Pflegeversicherung | 1995 | 5 levels (2021):  €316 – €901 *(level 2-5)*  *Note: In Germany, care level 1 (Pflegegrad 1) offers limited cash benefits compared to higher care levels, as it is intended for individuals with minor impairments in daily living. They can however claim relief allowance of up to €125 per month to compensate for expenses for outpatient care services for nursing care at home.* | Yes | Yes | No |
| *Note*: *Information gathered from varying sources, including MISSOC 2022; Gateway to Global Aging Data 2024; Ledoux und Dussuet 2020; Ariaans, Linden, und Wendt 2021; Ministère des Solidarités et de la Santé 2025* | | | | | | |

Table A.7: Out-of-pocket (OOP) payments for home care in selected European countries (SHARE Waves 6 and 7)

| Country | Wave | % Using OOP | Mean OOP | SD |
| --- | --- | --- | --- | --- |
| *Austria* | 6 | 71.3% | 3166.88 | 5845.54 |
| *Germany* | 6 | 41.4% | 1992.77 | 4546.00 |
| *France* | 6 | 63.5% | 1597.88 | 3180.75 |
| *Belgium* | 6 | 82.4% | 1344.72 | 1980.22 |
| *Austria* | 7 | 65.3% | 2755.38 | 5191.65 |
| *Germany* | 7 | 57.3% | 1732.93 | 4063.90 |
| *France* | 7 | 62.2% | 4118.28 | 15446.30 |
| *Belgium* | 7 | 79.4% | 1310.89 | 1544.04 |

Note: *Data indicates whether an individual paid anything for home care without reimbursement from health or long-term care insurance (% using OOP), as well as the average amount paid for professional help — including personal care, meals-on-wheels, or other domestic tasks— over the past twelve months.*

**Appendix B**

**Regression results**

Table B.1: Regression results on mixed and no care use (GLM vs IV-GLM comparisons)

|  | **Dependent variable:** | | | | | | | |
| --- | --- | --- | --- | --- | --- | --- | --- | --- |
|  |  | | | | | | | |
|  | **Mixed care** | | | | **No care** | | | |
|  | ***GLM*** | | ***IV-GLM*** | | ***GLM*** | | ***IV-GLM*** | |
|  | **(1)** | **(2)** | **(3)** | **(4)** | **(5)** | **(6)** | **(7)** | **(8)** |
| **age** | 0.018^***^ | 0.018^***^ | 0.018^**^ | 0.021^***^ | -0.018^***^ | -0.018^***^ | -0.018^***^ | -0.017^***^ |
|  | (0.005) | (0.005) | (0.004) | (0.005) | (0.003) | (0.003) | (0.003) | (0.003) |
|  |  |  |  |  |  |  |  |  |
| **iadl** | 0.236^***^ | 0.239^***^ | 0.236^***^ | 0.241^***^ | -0.215^***^ | -0.219^***^ | -0.215^***^ | -0.215^***^ |
|  | (0.021) | (0.021) | (0.021) | (0.021) | (0.014) | (0.014) | (0.013) | (0.013) |
|  |  |  |  |  |  |  |  |  |
| **adl** | 0.297^***^ | 0.298^***^ | 0.297^***^ | 0.297^***^ | -0.430^***^ | -0.430^***^ | -0.428^***^ | -0.429^***^ |
|  | (0.026) | (0.027) | (0.026) | (0.026) | (0.024) | (0.024) | (0.021) | (0.023) |
|  |  |  |  |  |  |  |  |  |
| **female** (ref. male) | 0.152^*^ | 0.155^*^ | 0.152^*^ | 0.156* | -0.029 | -0.034 | -0.029 | -0.030 |
|  | (0.090) | (0.090) | (0.089) | (0.089) | (0.053) | (0.053) | (0.052) | (0.052) |
|  |  |  |  |  |  |  |  |  |
| **children** (ref. none) | 0.223 | 0.217 | 0.223^*^ | 0.230 | -0.219^***^ | -0.208^**^ | -0.223^**^ | -0.223^**^ |
|  | (0.136) | (0.136) | (0.129) | (0.136) | (0.083) | (0.082) | (0.082) | (0.082) |
|  |  |  |  |  |  |  |  |  |
| **spouse** (ref. none) | 0.259^**^ | 0.251^**^ | 0.257^***^ | 0.264^***^ | -0.469^***^ | -0.471^***^ | -0.466^***^ | -0.469^***^ |
|  | (0.101) | (0.101) | (0.101) | (0.101) | (0.058) | (0.058) | (0.057) | (0.057) |
|  |  |  |  |  |  |  |  |  |
| **wealth quintiles** (ref: 1^st^ quintile) |  |  |  |  |  |  |  |  |
| *2^nd^ quintile* | 0.178 | 0.189 | 0.168 | 0.128 | -0.057 | -0.064 | -0.035 | -0.068 |
|  | (0.116) | (0.116) | (0.117) | (0.124) | (0.070) | (0.070) | (0.071) | (0.076) |
|  |  |  |  |  |  |  |  |  |
| *3^rd^ quintile* | 0.190 | 0.192 | 0.178 | 0.132 | -0.124^*^ | -0.133^*^ | -0.097 | -0.139 |
|  | (0.129) | (0.129) | (0.131) | (0.142) | (0.075) | (0.075) | (0.077) | (0.085) |
|  |  |  |  |  |  |  |  |  |
| *4^th^ quintile* | 0.247^*^ | 0.242^*^ | 0.235 | 0.190 | 0.013 | 0.013 | 0.041 | -0.006 |
|  | (0.136) | (0.137) | (0.139) | (0.151) | (0.081) | (0.081) | (0.083) | (0.092) |
|  |  |  |  |  |  |  |  |  |
| *5^th^ quintile* | 0.062 | 0.056 | 0.047 | -0.014 | -0.004 | -0.009 | 0.028 | -0.026 |
|  | (0.160) | (0.161) | (0.162) | (0.176) | (0.090) | (0.091) | (0.093) | (0.104) |
|  |  |  |  |  |  |  |  |  |
| **education** (years) | -0.013 | -0.014 | -0.013 | -0.013 | 0.021^***^ | 0.022^***^ | 0.019^***^ | 0.020^***^ |
|  | (0.012) | (0.012) | (0.011) | (0.011) | (0.007) | (0.007) | (0.006) | (0.006) |
|  |  |  |  |  |  |  |  |  |
| **CFC receipt** (ref. non-receipt) | 0.516^***^ | 0.364^*^ | 0.292 | -0.176 | -0.872^***^ | -0.696^***^ | -0.335 | -0.174 |
|  | (0.133) | (0.188) | (0.535) | (0.561) | (0.099) | (0.132) | (0.383) | (0.392) |
|  |  |  |  |  |  |  |  |  |
| **country** (ref. *Austria*) |  |  |  |  |  |  |  |  |
| *Belgium* | -0.153 | -0.194 | -0.202 | 0.158 | -0.041 | -0.012 | 0.071 | 0.140 |
|  | (0.138) | (0.149) | (0.178) | (0.242) | (0.080) | (0.081) | (0.108) | (0.155) |
|  |  |  |  |  |  |  |  |  |
| *France* | -0.249^*^ | -0.330^**^ | -0.297 | -0.329 | 0.206^**^ | 0.273^***^ | 0.314 | 0.453^***^ |
|  | (0.145) | (0.159) | (0.181) | (0.274) | (0.084) | (0.088) | (0.109) | (0.148) |
|  |  |  |  |  |  |  |  |  |
| *Germany* | 0.674^***^ | 0.539^***^ | 0.63^***^ | 0.231 | -0.135 | -0.034 | -0.033 | 0.203 |
|  | (0.132) | (0.150) | (0.168) | (0.216) | (0.083) | (0.088) | (0.106) | (0.137) |
|  |  |  |  |  |  |  |  |  |
| **CFC × *Belgium*** |  | -0.737 |  | -10.71^**^ |  | 1.207^***^ |  | -0.687 |
|  |  | (0.517) |  | (4.26) |  | (0.437) |  | (3.19) |
|  |  |  |  |  |  |  |  |  |
| **CFC × *France*** |  | 0.204 |  | -1.079 |  | -0.475^*^ |  | -2.08 |
|  |  | (0.435) |  | (3.8) |  | (0.269) |  | (2.02) |
|  |  |  |  |  |  |  |  |  |
| **CFC × *Germany*** |  | 0.574^**^ |  | 5.001^***^ |  | -0.925^***^ |  | -3.341^***^ |
|  |  | (0.279) |  | (1.75) |  | (0.253) |  | (1.27) |
|  |  |  |  |  |  |  |  |  |
|  |  |  |  |  |  |  |  |  |
| **Observations** | 10,283 | 10,283 | 10,283 | 10,283 | 10,283 | 10,283 | 10,283 | 10,283 |
| **Wave FE** | Yes | Yes | Yes | Yes | Yes | Yes | Yes | Yes |
| *Note: Coefficients displayed as log-odds; Standard errors in brackets* ^*^p<0.1; ^**^p<0.05; ^***^p<0.01 | | | | | | | | |

**Robustness & sensitivity**

Table B. 2: Regression results on informal and formal care use (GLM vs IV-GLM comparisons based on the reduced sample of individuals observed at least twice)

|  | **Dependent variable:** | | | | | | | |
| --- | --- | --- | --- | --- | --- | --- | --- | --- |
|  |  | | | | | | | |
|  | **Informal care** | | | | **Formal care** | | | |
|  | ***GLM*** | | ***IV-GLM*** | | ***GLM*** | | ***IV-GLM*** | |
|  | **(1)** | **(2)** | **(3)** | **(4)** | **(5)** | **(6)** | **(7)** | **(8)** |
| **age** | -0.002 | -0.001 | -0.003 | 0.001 | 0.038^***^ | 0.038^***^ | 0.040^***^ | 0.037^***^ |
|  | (0.004) | (0.004) | (0.004) | (0.004) | (0.005) | (0.005) | (0.004) | (0.0087 |
|  |  |  |  |  |  |  |  |  |
| **iadl** | 0.142^***^ | 0.145^***^ | 0.140^***^ | 0.146^***^ | 0.179^***^ | 0.179^***^ | 0.181^***^ | 0.181^***^ |
|  | (0.018) | (0.018) | (0.018) | (0.018) | (0.020) | (0.020) | (0.020) | (0.020) |
|  |  |  |  |  |  |  |  |  |
| **adl** | 0.332^***^ | 0.334^***^ | 0.332^***^ | 0.333^***^ | 0.223^***^ | 0.223^***^ | 0.219^***^ | 0.218^***^ |
|  | (0.027) | (0.027) | (0.027) | (0.028) | (0.028) | (0.028) | (0.029) | (0.028) |
|  |  |  |  |  |  |  |  |  |
| **female** (ref. male) | -0.111 | -0.105 | -0.108 | -0.115 | 0.216^**^ | 0.217^**^ | 0.214^**^ | 0.224^**^ |
|  | (0.074) | (0.075) | (0.074) | (0.074) | (0.091) | (0.091) | (0.091) | (0.090) |
|  |  |  |  |  |  |  |  |  |
| **children** (ref. none) | 0.405^***^ | 0.387^***^ | 0.399^***^ | 0.409^***^ | -0.201^*^ | -0.202^*^ | -0.197* | -0.195* |
|  | (0.113) | (0.114) | (0.117) | (0.117) | (0.114) | (0.115) | (0.117) | (0.117) |
|  |  |  |  |  |  |  |  |  |
| **spouse** (ref. none) | 1.017^***^ | 1.029^***^ | 1.021^***^ | 1.046^***^ | -0.481^***^ | -0.487^***^ | -0.484^***^ | -0.484^***^ |
|  | (0.083) | (0.083) | (0.084) | (0.085) | (0.093) | (0.094) | (0.096) | (0.096) |
|  |  |  |  |  |  |  |  |  |
| **wealth quintiles** (ref: 1^st^ quintile) |  |  |  |  |  |  |  |  |
| *2^nd^ quintile* | 0.161^*^ | 0.170^*^ | 0.190 | 0.136 | 0.053 | 0.050 | 0.001 | 0.047 |
|  | (0.094) | (0.095) | (0.096) | (0.107) | (0.105) | (0.105) | (0.112) | (0.121) |
|  |  |  |  |  |  |  |  |  |
| *3^rd^ quintile* | 0.111 | 0.116 | 0.145 | 0.079 | 0.108 | 0.104 | 0.045 | 0.102 |
|  | (0.104) | (0.104) | (0.109) | (0.12) | (0.119) | (0.119) | (0.123) | (0.136) |
|  |  |  |  |  |  |  |  |  |
| *4^th^ quintile* | -0.024 | -0.023 | 0.007 | -0.052 | -0.055 | -0.068 | -0.112 | -0.060 |
|  | (0.116) | (0.117) | (0.12) | (0.127) | (0.139) | (0.139) | (0.145) | (0.152) |
|  |  |  |  |  |  |  |  |  |
| *5^th^ quintile* | 0.133 | 0.134 | 0.180 | 0.090 | -0.292^*^ | -0.301^*^ | -0.387^**^ | -0.309 |
|  | (0.125) | (0.125) | (0.129) | (0.146) | (0.163) | (0.163) | (0.175) | (0.192) |
|  |  |  |  |  |  |  |  |  |
| **education** (years) | -0.014 | -0.015 | -0.014 | -0.014 | -0.009 | -0.008 | -0.007 | -0.008 |
|  | (0.009) | (0.009) | (0.009) | (0.009) | (0.011) | (0.011) | (0.011) | (0.011) |
|  |  |  |  |  |  |  |  |  |
| **formal care** | 1.025^***^ | 1.028^***^ | 1.034^***^ | 1.034^***^ |  |  |  |  |
|  | (0.090) | (0.090) | (0.095) | (0.096) |  |  |  |  |
|  |  |  |  |  |  |  |  |  |
| **informal care** |  |  |  |  | 1.098^***^ | 1.101^***^ | 1.108^***^ | 1.104^***^ |
|  |  |  |  |  | (0.089) | (0.089) | (0.092) | (0.092) |
|  |  |  |  |  |  |  |  |  |
| **CFC receipt** (ref. non-receipt) | 0.641^***^ | 0.713^***^ | 1.325^***^ | 0.993^**^ | 0.272^**^ | 0.067 | -1.116^**^ | -1.385^**^ |
|  | (0.117) | (0.163) | (0.455) | (0.474) | (0.133) | (0.195) | (0.539) | (0.557) |
|  |  |  |  |  |  |  |  |  |
| **country** (ref. *Austria*) |  |  |  |  |  |  |  |  |
| *Belgium* | -0.482^***^ | -0.410^***^ | -0.312^**^ | 0.094 | 0.702^***^ | 0.594^***^ | 0.335^*^ | 0.126 |
|  | (0.109) | (0.119) | (0.155) | (0.215) | (0.137) | (0.154) | (0.193) | (0.273) |
|  |  |  |  |  |  |  |  |  |
| *France* | -0.306^***^ | -0.310^**^ | -0.14 | -0.424^*^ | 0.097 | 0.017 | -0.262 | -0.395 |
|  | (0.116) | (0.128) | (0.157) | (0.224) | (0.151) | (0.169) | (0.201) | (0.296) |
|  |  |  |  |  |  |  |  |  |
| *Germany* | -0.027 | -0.008 | 0.128 | -0.111 | 0.812^***^ | 0.658^***^ | 0.477^**^ | 0.158 |
|  | (0.115) | (0.128) | (0.15) | (0.196) | (0.142) | (0.166) | (0.188) | (0.237) |
|  |  |  |  |  |  |  |  |  |
| **CFC × *Belgium*** |  | -1.686^***^ |  | -11.5^***^ |  | 0.352 |  | 2.63 |
|  |  | (0.441) |  | (3.69) |  | (0.411) |  | (4.39) |
|  |  |  |  |  |  |  |  |  |
| **CFC × *France*** |  | 0.535 |  | 4.053 |  | -0.245 |  | 1.042 |
|  |  | (0.358) |  | (2.92) |  | (0.485) |  | (3.6) |
|  |  |  |  |  |  |  |  |  |
| **CFC × *Germany*** |  | 0.062 |  | 2.522^*^ |  | 0.557^*^ |  | 3.15^**^ |
|  |  | (0.281) |  | (1.52) |  | (0.298) |  | (1.55) |
|  |  |  |  |  |  |  |  |  |
|  |  |  |  |  |  |  |  |  |
| **Observations** | 5,834 | 5,834 | 5,834 | 5,834 | 5,834 | 5,834 | 5,834 | 5,834 |
| **Wave FE** | Yes | Yes | Yes | Yes | Yes | Yes | Yes | Yes |
| *Note*: *Coefficients displayed as log-odds; Standard errors in brackets* ^*^p<0.1; ^**^p<0.05; ^***^p<0.01 | | | | | | | | |

**IV Validity**

Table B. 3: IV-GLM results on informal and formal care use accounting for wave and country FE in the first stage

|  | ***Dependent variable:*** | | | | |  |  |
| --- | --- | --- | --- | --- | --- | --- | --- |
|  |  | | | | |  |  |
|  | **Informal care** | | **Formal care** | | |  |  |
|  | **(1)** | **(2)** |  | **(3)** | **(4)** |  |  |
| **age** | -0.004 | -0.001 |  | 0.041^***^ | 0.052^***^ |  |  |
|  | (0.003) | (0.005) |  | (0.004) | (0.008) |  |  |
|  |  |  |  |  |  |  |  |
| **iadl** | 0.148^***^ | 0.151^***^ |  | 0.171^***^ | 0.174^***^ |  |  |
|  | (0.015) | (0.015) |  | (0.016) | (0.017) |  |  |
|  |  |  |  |  |  |  |  |
| **adl** | 0.323^***^ | 0.325^***^ |  | 0.224^***^ | 0.224^***^ |  |  |
|  | (0.022) | (0.022) |  | (0.024) | (0.024) |  |  |
|  |  |  |  |  |  |  |  |
| **female** (ref. male) | -0.059 | -0.050 |  | 0.166^**^ | 0.183^**^ |  |  |
|  | (0.057) | (0.057) |  | (0.072) | (0.083) |  |  |
|  |  |  |  |  |  |  |  |
| **children** (ref. none) | 0.364^***^ | 0.371^***^ |  | -0.083 | -0.081 |  |  |
|  | (0.094) | (0.094) |  | (0.098) | (0.097) |  |  |
|  |  |  |  |  |  |  |  |
| **spouse** (ref. none) | 0.988^***^ | 0.992^***^ |  | -0.576^***^ | -0.572^***^ |  |  |
|  | (0.068) | (0.068) |  | (0.079) | (0.079) |  |  |
| **wealth quintiles** (ref: 1^st^ quintile) |  |  |  |  |  |  |  |
| *2^nd^ quintile* | 0.078 | 0.036 |  | -0.012 | -0.201 |  |  |
|  | (0.082) | (0.103) |  | (0.095) | (0.159) |  |  |
|  |  |  |  |  |  |  |  |
| *3^rd^ quintile* | 0.093 | 0.039 |  | 0.049 | -0.195 |  |  |
|  | (0.089) | (0.12) |  | (0.105) | (0.192) |  |  |
|  |  |  |  |  |  |  |  |
| *4^th^ quintile* | 0.035 | -0.022 |  | -0.043 | -0.310 |  |  |
|  | (0.096) | (0.131) |  | (0.117) | (0.207) |  |  |
|  |  |  |  |  |  |  |  |
| *5^th^ quintile* | 0.069 | 0.003 |  | -0.174 | -0.485^**^ |  |  |
|  | (0.106) | (0.146) |  | (0.139) | (0.235) |  |  |
|  |  |  |  |  |  |  |  |
| **education** (years) | -0.019^**^ | -0.018^**^ |  | -0.009 | -0.007 |  |  |
|  | (0.007) | (0.007) |  | (0.009) | (0.011) |  |  |
|  |  |  |  |  |  |  |  |
| **formal care** | 1.153^***^ | 1.146^***^ |  |  |  |  |  |
|  | (0.075) | (0.076) |  |  |  |  |  |
|  |  |  |  |  |  |  |  |
| **informal care** |  |  |  | 1.245^***^ | 1.239^***^ |  |  |
|  |  |  |  | (0.073) | (0.073) |  |  |
| **CFC receipt** (ref. non-receipt) | 1.516^**^ | 0.736 |  | -1.638^**^ | -2.864^***^ |  |  |
|  | (0.646) | (0.768) |  | (0.739) | (1.1) |  |  |
| **country** (ref. *Austria*) |  |  |  |  |  |  |  |
| *Belgium* | -0.165 | -0.058 |  | 0.012 | 0.217 |  |  |
|  | (0.184) | (0.199) |  | (0.226) | (0.258) |  |  |
|  |  |  |  |  |  |  |  |
| *France* | -0.148 | -0.407^*^ |  | -0.423^*^ | -0.313 |  |  |
|  | (0.173) | (0.213) |  | (0.217) | (0.284) |  |  |
|  |  |  |  |  |  |  |  |
| *Germany* | 0.101 | -0.362^*^ |  | 0.245 | 0.132 |  |  |
|  | (0.155) | (0.213) |  | (0.192) | (0.275) |  |  |
|  |  |  |  |  |  |  |  |
| **CFC × *Belgium*** |  | -19.5^*^ |  |  | -28.31^*^ |  |  |
|  |  | (10.2) |  |  | (15.1) |  |  |
|  |  |  |  |  |  |  |  |
| **CFC × *France*** |  | -1.811 |  |  | -8.424 |  |  |
|  |  | (3.85) |  |  | (5.37) |  |  |
|  |  |  |  |  |  |  |  |
| **CFC × *Germany*** |  | 4.198^*^ |  |  | -1.323 |  |  |
|  |  | (2.15) |  | | (2.82) |  |  |
| **Observations** | 10,283 | 10,283 |  | 10,283 | 10,283 |  |  |
| **Wave FE** | Yes | Yes |  | Yes | Yes |  |  |
| *Note*: *Coefficients displayed as log-odds; Standard errors in brackets* ^*^p<0.1; ^**^p<0.05; ^***^p<0.01 | | | | | | |  |
|  | | | | | | | |

Table B.4: IV-GLM results on informal and formal care using a modified instrument (granting generosity calculated at wave, country, and NUTS-1 level)

|  | ***Dependent variable:*** | | | | | | | |  |
| --- | --- | --- | --- | --- | --- | --- | --- | --- | --- |
|  |  | | | | | | | |  |
|  | **Informal care** | | **Formal care** | | **Mixed care** | | **No care** | |  |
|  | **(1)** | **(2)** | **(3)** | **(4)** | **(5)** | **(6)** | **(7)** | **(8)** |  |
| **CFC receipt** | 0.965^*^ | 0.355 | -0.715 | -1.085 | 1.106 | -0.066 | -0.022 | 0.308 |  |
|  | (0.545) | (0.575) | (0.630) | (0.668) | (0.770) | (0.794) | (0.522) | (0.545) |  |
| **country** (ref. *Austria*) |  |  |  |  |  |  |  |  |  |
| *Belgium* | -0.318^**^ | -0.190 | -0.315^*^ | 0.247 | -0.006 | 0.224 | 0.159 | 0.274 |  |
|  | (0.148) | (0.180) | (0.184) | (0.231) | (0.229) | (0.274) | (0.139) | (0.179) |  |
| *France* | -0.282^*^ | -0.517^**^ | -0.167 | -0.071 | -0.107 | 0.036 | 0.400^***^ | 0.610^***^ |  |
|  | (0.147) | (0.200) | (0.186) | (0.253) | (0.228) | (0.316) | (0.139) | (0.186) |  |
| *Germany* | -0.009 | -0.313^*^ | 0.440^**^ | 0.220 | 0.806^***^ | 0.309 | 0.046 | 0.317^*^ |  |
|  | (0.943) | (0.172) | (0.175) | (0.308) | (0.213) | (0.254) | (0.133) | (0.162) |  |
| **CFC × *Belgium*** |  | -8.161^**^ |  | -0.659 |  | -13.56^**^ |  | -0.846 |  |
|  |  | (3.753) |  | (0.887) |  | (5.481) |  | (4.13) |  |
| **CFC × *France*** |  | 2.369 |  | -3.803 |  | -8.656^*^ |  | -3.027 |  |
|  |  | (3.446) |  | (3.957) |  | (5.145) |  | (3.16) |  |
| **CFC × *Germany*** |  | 3.201^**^ |  | 2.237 |  | 4.318^**^ |  | -3.385^***^ |  |
|  |  | (1.286) |  | (1.395) |  | (1.609) |  | (1.175) |  |
|  |  |  |  |  |  |  |  |  |  |
|  |  |  |  |  |  |  |  |  |  |
| **Observations** | 10,283 | 10,283 | 10,283 | 10,283 | 10,283 | 10,283 | 10,283 | 10,283 |  |
| **Covariates** | Yes | Yes | Yes | Yes | Yes | Yes | Yes | Yes |  |
| **Wave FE** | Yes | Yes | Yes | Yes | Yes | Yes | Yes | Yes |  |
| *Note: Coefficients displayed as log-odds; Standard errors in brackets* ^*^p<0.1; ^**^p<0.05; ^***^p<0.01 | | | | | | | | | |

Table B.5: IV-GLM results on informal and formal care using the restricted sample including Austria, France, and Germany

|  | ***Dependent variable:*** | | | | | | | |  |
| --- | --- | --- | --- | --- | --- | --- | --- | --- | --- |
|  |  | | | | | | | |  |
|  | **Informal care** | | **Formal care** | | **Mixed care** | | **No care** | |  |
|  | **(1)** | **(2)** | **(3)** | **(4)** | **(5)** | **(6)** | **(7)** | **(8)** |  |
| **CFC receipt** | 0.976^**^ | 0.887^*^ | -1.303^**^ | -1.407^**^ | 0.144 | -0.097 | -0.068 | 0.051 |  |
|  | (0.478) | (0.482) | (0.589) | (0.595) | (0.655) | (0.66) | (0.471) | (0.475) |  |
| **country** (ref. *Austria*) |  |  |  |  |  |  |  |  |  |
| *France* | -0.258^**^ | -0.398^**^ | -0.198 | -0.141 | -0.303 | -0.201 | 0.331^***^ | 0.493^***^ |  |
|  | (0.124) | (0.097) | (0.161) | (0.242) | (0.194) | (0.305) | (0.116) | (0.167) |  |
|  |  |  |  |  |  |  |  |  |  |
| *Germany* | -0.011 | -0.278^*^ | 0.412^***^ | -0.235 | 0.658^***^ | 0.241 | 0.014 | 0.297^*^ |  |
|  | (0.12) | (0.166) | (0.153) | (0.212) | (0.179) | (0.245) | (0.113) | (0.157) |  |
|  |  |  |  |  |  |  |  |  |  |
| **CFC × *France*** |  | 1.763 |  | -1.044 |  | -2.073 |  | -2.005 |  |
|  |  | (1.99) |  | (2.51) |  | (3.4) |  | (1.82) |  |
|  |  |  |  |  |  |  |  |  |  |
| **CFC × *German*y** |  | 3.15^**^ |  | 1.871 |  | 4.324^**^ |  | -3.26^**^ |  |
|  |  | (1.38) |  | (1.57) |  | (1.82) |  | (1.27) |  |
|  |  |  |  |  |  |  |  |  |  |
|  |  |  |  |  |  |  |  |  |  |
| **Observations** | 10,283 | 10,283 | 10,283 | 10,283 | 10,283 | 10,283 | 10,283 | 10,283 |  |
| **Covariates** | Yes | Yes | Yes | Yes | Yes | Yes | Yes | Yes |  |
| **Wave FE** | Yes | Yes | Yes | Yes | Yes | Yes | Yes | Yes |  |
| *Note*: *Coefficients displayed as log-odds; Standard errors in brackets* ^*^p<0.1; ^**^p<0.05; ^***^p<0.01 | | | | | | | | | |

Table B.6: Falsification test (Subsample: non-benefit receivers)

|  | ***Dependent variable:*** | | | | |  |  |
| --- | --- | --- | --- | --- | --- | --- | --- |
|  | | **Informal care** | **Formal care** | **Mixed care** | **No care** | |  |
|  | | **(1)** | **(2)** | **(3)** | **(4)** | |  |
| **granting generosity** | | 0.209 | 0.519 | -0.785 | 0.502 | |  |
|  | | (0.597) | (0.747) | (0.619) | (0.747) | |  |
| **Observations** | | 9,418 | 9,418 | 9,418 | 9,418 | |  |
| **Covariates** | | Yes | Yes | Yes | Yes | |  |
| **Country FE** | | Yes | Yes | Yes | Yes | |  |
| **Wave FE** | | Yes | Yes | Yes | Yes | |  |
| *Note: Coefficient displayed as log-odds; Standard errors in brackets* ^*^p<0.1; ^**^p<0.05; ^***^p<0.01 | | | | | | | |

Table B.7: Monotonicity test across varying age groups

|  | ***Dependent variable:*** | | |
| --- | --- | --- | --- |
|  |  | | |
|  | **CFC receipt** (ref. non-receipt) | | |
|  | *< 65 years* | *65-80 years* | *80+ years* |
|  | **(1)** | **(2)** | **(3)** |
|  | | | |
| **granting generosity** | 4.480^**^ | 2.959^***^ | 3.039^***^ |
|  | (2.061) | (0.891) | (0.700) |
|  |  |  |  |
| **Observations** | 1,898 | 4,075 | 3,742 |
| **Covariates** | Yes | Yes | Yes |
| **Country FE** | Yes | Yes | Yes |
| **Wave FE** | Yes | Yes | Yes |
|  | | | |
| *Note: Coefficient displayed as log-odds; Standard errors in brackets* ^*^p<0.1; ^**^p<0.05; ^***^p<0.01 | | | |

Table B.8: IV-GLM results (adding average country-wave benefit amount)

|  | ***Dependent variable:*** | |
| --- | --- | --- |
|  | **Informal care** | **Formal care** |
|  | **(1)** | **(2)** |
|  | | |
| **CFC receipt** | 1.125^***^ | -0.866^*^ |
|  | (0.395) | (0.471) |
| Average benefit amount (by country/wave) | -0.0002 | -0.001 |
|  | (0.0003) | (0.0004) |
| **Observations** | 10,283 | 10,283 |
| **Covariates** | Yes | Yes |
| **Country FE** | Yes | Yes |
| **Wave FE** | Yes | Yes |
| *Note: Average amounts based on non-missing cases in SHARE ep_078 variable* | ^*^p<0.1; ^**^p<0.05; ^***^p<0.01 | |

Table B.9: IV-GLM results on informal and formal care (adding country-wave interactions)

|  | ***Dependent variable:*** | | | |
| --- | --- | --- | --- | --- |
|  | **Informal care** | | | **Formal care** |
|  | **(1)** | | | **(2)** |
|  | | | | |
| **CFC receipt** | 1.157^***^ | | | -0.699 |
|  | (0.432) | | | (0.507) |
| **Country** (ref. *Austria*) |  | | |  |
|  |  | | |  |
| *Belgium* | -0.229^*^ | | | 0.132 |
|  | (0.132) | | | (0.171) |
| *France* | -0.315^**^ | | | -0.350 |
|  | (0.140) | | | (0.186) |
| *Germany* | -0.037 | | | 0.723^***^ |
|  | (0.138) | | | (0.170) |
| **Wave** (ref. *Wave 6*) |  | | |  |
| *Wave 7* | 0.017 | | | -0.250 |
|  | (0.245) | | | (0.335) |
| *Wave 8* | -0.055 | | | -0.251 |
|  | (0.167) | | | (0.210) |
| *Wave 9* | -0.205 | | | -0.184 |
|  | (0.144) | | | (0.182) |
| **Country *× Wave*** |  | | |  |
| Belgium × *wave 7* | -0.028 | | | -0.379 |
|  | (0.285) | | | (0.376) |
| France × *wave 7* | 0.175 | | | 0.262 |
|  | (0.311) | | | (0.419) |
| Germany × *wave 7* | 0.088 | | | 0.290 |
|  | (0.318) | | | (0.400) |
| Belgium × *wave 8* | -0.135 | | | 0.476^*^ |
|  | (0.221) | | | (0.262) |
| France × *wave 8* | 0.032 | | | 0.458 |
|  | (0.222) | | | (0.280) |
| Germany × *wave 8* | -0.230 | | | -0.298 |
|  | (0.219) | | | (0.262) |
| Belgium × *wave 9* | -0.097 | | | 0.350 |
|  | (0.197) | | | (0.227) |
| France × *wave 9* | 0.098 | | | 0.416 |
|  | (0.227) | | | (0.271) |
| Germany × *wave 9* | 0.309 | | | -0.573^**^ |
|  | (0.200) | | | (0.238) |
| **Observations** | | 10,283 | 10,283 | |
| **Covariates** | Yes | | | Yes |
|  | ^*^p<0.1; ^**^p<0.05; ^***^p<0.01 | | | |

1. ‘*Yes’* may denote that the granting of care allowance or benefit amount is income- or property-related.
2. For instance., the amount of the allowance for assistance to the elderly may be reduced by the income of the disabled person and of the person with whom s/he forms a household, which exceeds certain ceilings.
3. In addition, the **disability compensation allowance** (PCH) can be claimed at any age if the eligibility conditions are fulfilled before the age of 60 (or if the person concerned continues to work).
4. No earlier information publicly available.
5. Use is defined by a predetermined care package defined by the family and/or professionals.
